# Effect of imidafenacin on the urodynamic parameters of patients with indwelling bladder catheters due to spinal cord injury

**DOI:** 10.1038/sc.2016.168

**Published:** 2016-11-29

**Authors:** H Sugiyama, O Uemura, T Mori, N Okisio, K Unai, M Liu

**Affiliations:** 1 National Hospital Organization Murayama Medical Center, Tokyo, Japan; 2 Department of Rehabilitation Medicine, Keio University School of Medicine, Tokyo, Japan

## Abstract

**Study design:**

A retrospective study.

**Objectives:**

To investigate the effect of imidafenacin on the urodynamic parameters of patients with indwelling bladder catheters due to spinal cord injury (SCI).

**Setting:**

Spinal center (Tokyo, Japan).

**Methods:**

Imidafenacin was prescribed to 34 patients with SCI who had a low cystometric volume and/or detrusor compliance according to a urodynamic study. A low cystometric volume and detrusor compliance were defined as <200 ml and <20 ml cm^−1^ H_2_O, respectively. The urodynamic study was repeated 4 weeks after imidafenacin was prescribed. When the urodynamic parameters did not improve in the follow-up study, the dose of imidafenacin was increased twofold. Then the urodynamic study was repeated 4 weeks thereafter. We compared the urodynamic parameters before and after imidafenacin treatment. Complications such as vesico-urethral reflux (VUR) and autonomic dysreflexia (AD) were documented.

**Results:**

Fifteen patients took 0.2 mg of imidafenacin daily, and 19 received 0.4 mg of imidafenacin daily. Imidafenacin increased the cystometric volume from 246.0 to 321.5 ml (median, *P*=0.002), detrusor compliance from 6.67 ml cm^−1^ H_2_O to 8.98 ml cm^−1^ H_2_O (median, *P*=0.012), and decreased the detrusor pressure from 37.0 cm H_2_O to 30.5 cm H_2_O (median, *P*=0.056). All three patients who had VUR fully recovered. Although 3 of 12 patients recovered from AD, 3 patients newly developed symptoms of AD. No patient withdrew from treatment due to adverse effects.

**Conclusion:**

Imidafenacin is a safe drug that may improve the urodynamic parameters of patients with SCI, and it possibly alleviates bladder complications.

## Introduction

Patients with spinal cord injury (SCI) often have detrusor overactivity in combination with detrusor-sphincter dyssynergia.^1^ This pathological condition results in urological complications, for example, bladder deformity, a low bladder compliance and vesico-urethral reflux (VUR).^1^ Such urological complications are highly associated with the incidence of renal failure, which used to be the number one cause of death among patients with SCI.^1, 2^ Thanks to accumulated knowledge and the widespread understanding of bladder management, various treatment options are now available; thus, the mortality rate of SCI has markedly decreased.^2, 3^ Among these treatment options, clean intermittent catheterization (CIC) is considered most appropriate when it is applicable.^2, 4^ CIC, however, is not always recommended in those who have severely compromised hand function, cognitive dysfunctions, a bladder capacity <200 ml, severe spasticity, and limited assistance from caregivers.^2^ It should also be noted that CIC can still cause urethral trauma and genital infection.^5, 6^

The most frequent neurologic category of SCI is incomplete tetraplegia.^7^ In these patients, functional recovery can be observed first in the lower extremities and then in the bladder, followed by the upper extremities. Less than 1% of patients with SCI experience complete recovery during their hospital stay.^7^ Therefore, almost all patients with incomplete tetraplegia experience bladder and/or hand skill dysfunction to some extent when they are transferred to rehabilitation hospitals. Those who have bladder dysfunction and are unable to perform CIC are often managed with transurethral catheters. Although there are conflicting reports about renal function and the prevalence of urological complications,^8, 9^ the European Association of Urology's guideline does not recommend indwelling catheterization because of the risk of urinary tract infection and significant long-term complications.^10^ Therefore, efforts should be made to remove indwelling catheters in those who would recover adequate hand skills to use CIC.

Bladder compliance tends to decrease with time in patients with indwelling bladder catheters due to SCI, and low bladder compliance is highly associated with bladder complications.^11^ Anticholinergics can alleviate this condition. Kim *et al.*^12^ retrospectively analyzed patients with SCI and chronic indwelling catheters for bladder management with or without oxybutynin. They found that patients treated with oxybutynin showed favorable bladder leak point pressure and bladder compliance compared with those who were not treated with oxybutynin. Oxybutynin, however, has a high incidence of adverse events in a dose-dependent manner. Chapple *et al.*^13^ reported that oxybutynin's immediate-release formulation was not well tolerated in patients with overactive bladder. Although dry mouth is the most common adverse event and a leading cause of withdrawal in patients with overactive bladder, constipation can also be bothersome for those with SCI. Newer drugs with dose flexibility and/or formulation can subside these adverse effects.^14^

Imidafenacin is one of the newest anticholinergics that is selective of the urinary bladder, not the salivary glands.^15^ It has also been reported to have a significantly lower incidence of constipation than solifenacin, another new anti cholinergic agent, in patients with overactive bladder.^16^ As its approval for clinical use in Japan, many studies have affirmed its long-term safety, efficacy and tolerability.^17, 18^ However, it remains unknown whether imidafenacin alleviates bladder complications associated with SCI.

As the first step toward a randomized controlled trial in the future, we used retrospective data to determine the usefulness of imidafenacin for bladder management after SCI. Therefore, the current study aimed to assess the effects of imidafenacin, that is, whether it increases bladder capacity and compliance, and decreases detrusor pressure, which would lead to the future possibility of using CIC for bladder management in those with SCI.

## Materials and methods

We retrospectively reviewed the records of all patients who were treated with imidafenacin for neurogenic lower urinary tract dysfunction and had indwelling catheters due to SCI, and had undergone video urodynamic examination at our institution between July 2010 and December 2012. A chart review provided patient data on the injury level, completeness of injury, duration of injury and etiology. Patients taking other medications with antimuscarinic properties were excluded.

Demographic data are shown in Table 1. Thirty-four patients, 29 men and 5 women, were included. Patients' mean age was 60±15 years. The level of injury was the cervical spine in 26 patients and the thoracic spine in 8, and the injury was complete in 2 patients and incomplete in 32. The mean duration of injury was 102±45 days (range: 38–229 days). In most patients (*n*=20, 61%), the etiology of the injury was a fall.

The measurement and evaluation of urodynamic parameters were performed according to the current International Continence Society guidelines,^19^ and all patients gave written informed consent. Video urodynamic examination was performed with the patient in supine position. A 9-French triple lumen transurethral catheter and a rectal catheter were used to measure intravesical and urethral pressures, and abdominal pressure, respectively. The bladder was filled with a sterile saline solution containing a radiopaque contrast medium warmed to body temperature at a 50 ml min^−1^ filling rate. This solution was used for radiological analysis to assess bladder morphology and the presence of VUR. The intravesical, urethral and abdominal pressures were measured and recorded with standard urodynamic software (Life-Tech, Stafford, TX, USA). The filling of the bladder was stopped if one of the following conditions were met: (1) the infused volume reached 400 ml, (2) intravesical pressure reached 40 cm H_2_O, (3) patient expressed a maximum desire to void, (4) spontaneous urine leakage occurred, (5) VUR was present and (6) symptomatic autonomic dysreflexia (AD) was present, that is, the blood pressure increased due to headache, facial flushing, chills or sweating, and/or the systolic blood pressure increased >20 mm Hg.

**Figure 1:**
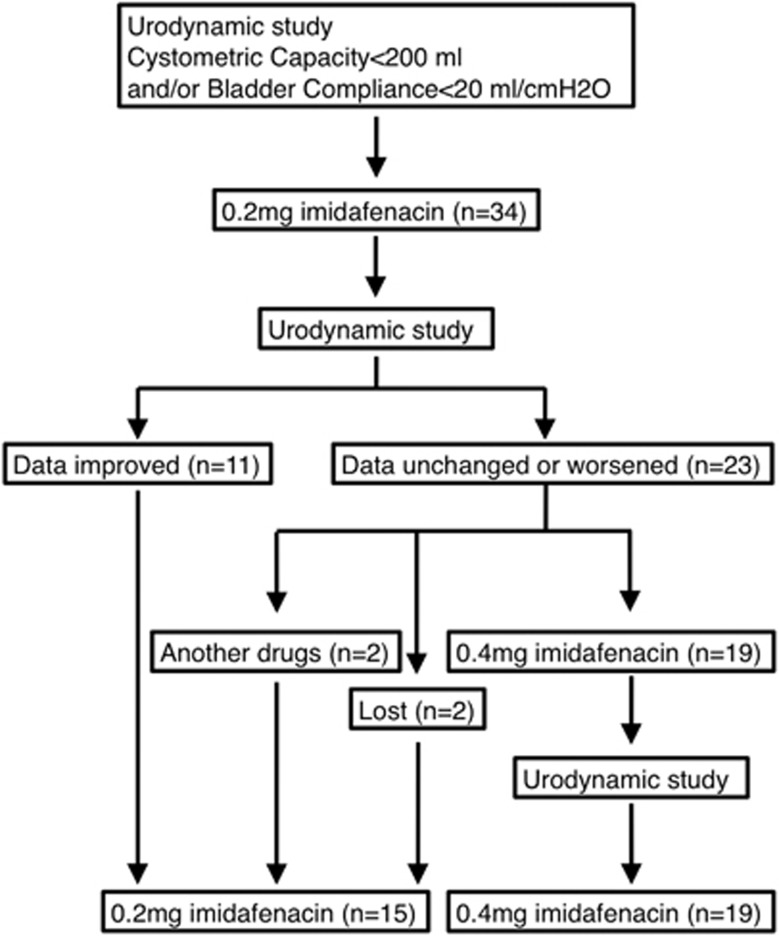
Trial procedure and patient flow chart.

The indications for imidafenacin treatment initiation and dosage escalation were a low cystometric capacity of <200 ml and/or low bladder compliance of <20 ml cm^−1^ H_2_O (Figure 1). Urodynamic data such as the cystometric capacity, maximum detrusor pressure during the filling phase and detrusor compliance before and after imidafenacin treatment were collected. The primary dosage of imidafenacin was 0.2 mg daily, and the follow-up data were collected at least 4 weeks after treatment initiation. The imidafenacin dosage was escalated to 0.4 mg daily, if a low cystometric capacity and/or low detrusor compliance remained at the first follow-up. During this treatment period, patients managed their bladder with transurethral catheters.

### Statistical analysis

Urodynamic data at baseline (preimidafenacin) and after treatment were compared. We evaluated the urodynamic data of patients who were prescribed 0.4 mg per day of imidafenacin at three times (at baseline, 4 weeks after the prescription and 4 weeks after the dosage escalation). The other patients were examined twice (at baseline and 4 weeks after the prescription). In all patients, we compared the baseline data and the last follow-up data. Wilcoxon signed-rank tests were used. A *P*-value <0.05 was considered significant. Statistical analyses were performed with SPSS software (version 22.0, IBM Corp., Armonk, NY, USA).

This study was approved by the institutional review board of Murayama Medical Center.

## Results

Thirty-four patients were evaluated, and 15 (44%) were treated with 0.2 mg of imidafenacin daily (Figure 1). Of them, 4 patients met the dosage escalation criteria, but they did not take 0.4 mg of imidafenacin daily (Figure 1). Two patients were lost to follow-up because of unexpected hospital discharge (Figure 1). The other two patients were prescribed other anticholinergics because their doctors inferred that they did not respond to imidafenacin treatment (Figure 1). Nineteen patients (56%) had dosage escalation and received 0.4 mg of imidafenacin daily at the final follow-up (Figure 1).

**Figure 2:**
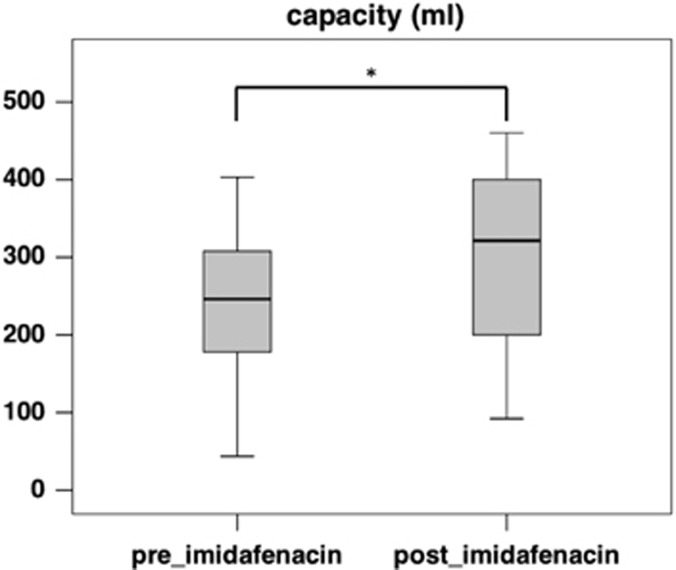
A box plot of bladder capacity before (preimidafenacin; median 246.0 ml, IQR 178.0–308.0 ml) and after (postimidafenacin; median 321.5 ml, IQR 200.0–400.0 ml, *P*=0.002) imidafenacin treatment (*n*=34). The asterisk (*) indicates a significant difference (Wilcoxon signed-rank test, *P*=0.002) between two values.

**Figure 3:**
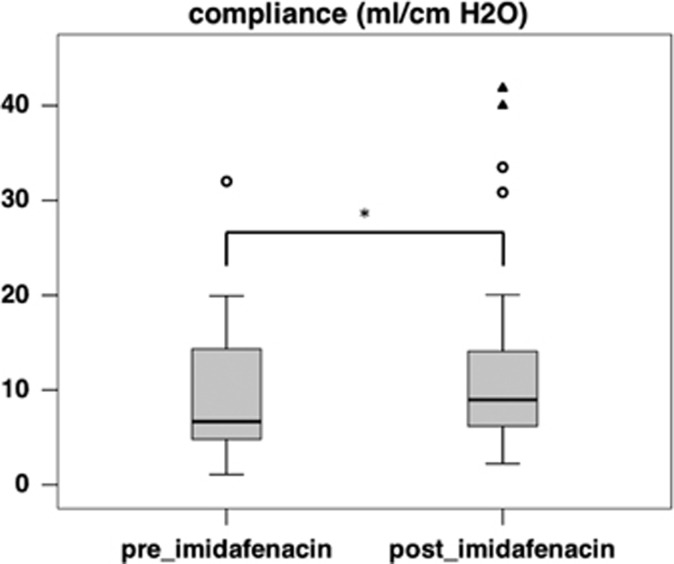
A box plot of detrusor compliance before (preimidafenacin; median 6.67 ml cm^−1^ H_2_O, IQR 4.81–14.32 ml cm^−1^ H_2_O) and after (postimidafenacin; median 8.98 ml per H_2_O, IQR 6.18–14.08 ml cm^−1^ H_2_O, *P*=0.012) imidafenacin treatment (*n*=34). The asterisk (*) indicates a significant difference (Wilcoxon signed-rank test, *P*=0.012) between two values.

**Figure 4:**
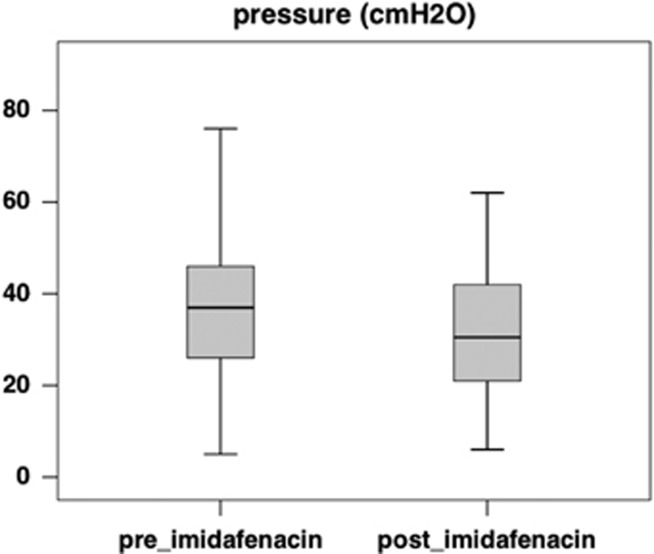
A box plot of maximum detrusor pressure before (preimidafenacin; median 37.0 cm H_2_O, IQR 26.0–46.0 cm H_2_O) and after (postimidafenacin; median 30.5 cm H_2_O, IQR 21.0–42.0 cm H_2_O) imidafenacin treatment (*n*=34). Note that this change was not statistically significant (Wilcoxon signed-rank test, *P*=0.056).

Imidafenacin treatment resulted in a significant improvement in the cystometric capacity (Figure 2), which increased from 246.0 ml (median, interquartile range (IQR) 178.0–308.0 ml) to 321.5 ml (median, IQR 200.0–400.0 ml, *P*=0.002), and detrusor compliance (Figure 3), which increased from 6.67 ml cm^−1^ H_2_O (median, IQR 4.81–14.32 ml cm^−1^ H_2_O) to 8.98 ml cm^−1^ H_2_O (median, IQR 6.18–14.08 ml cm^−1^ H_2_O, *P*=0.012). The maximum detrusor pressure during the filling phase decreased from 37.0 cm H_2_O (median, IQR 26.0–46.0 cm H_2_O) to 30.5 cm H_2_O (median, IQR 21.0–42.0 cm H_2_O, *P*=0.056), which was not statistically significant (Figure 4).

Before imidafenacin treatment, three patients presented with unilateral VUR, which was classified as grade I in 2 patients and grade II in 1 patient. All 3 patients fully recovered from VUR with imidafenacin treatment. Their cystometric volume increased from 200.0 ml (median, IQR 122.0–206.0 ml) to 310.0 ml (median, IQR 201.0–355.5 ml), and their detrusor compliance increased from 3.70 ml cm^−1^ H_2_O (median, IQR 2.40–5.93 ml cm^−1^ H_2_O) to 7.95 ml cm^−1^ H_2_O (median, IQR 5.10–14.00 ml cm^−1^ H_2_O). Symptomatic AD and/or an increase in blood pressure was documented in 12 patients (35%) before imidafenacin treatment. After imidafenacin treatment, 3 of 12 patients recovered, whereas the other 3 developed increased blood pressure during the follow-up period. Twelve patients (35%) were diagnosed as having symptomatic AD and/or increased blood pressure. The maximum detrusor pressure changed from 23.0 cm H_2_O (median, IQR 14.0–32.5 cm H_2_O) to 20.0 cm H_2_O (median, IQR 16.0–26.0 cm H_2_O) in those who recovered from AD, 40.0 cm H_2_O (median, IQR 27.0–50.0 cm H_2_O) to 29.0 cm H_2_O (median, IQR 27.0–55.0 cm H_2_O) in those who sustained AD, and 38.0 cm H_2_O (median, IQR 34.0–43.0 cm H_2_O) to 30.0 cm H_2_O (median, IQR 29.5–32.0 cm H_2_O) in those who newly acquired AD. No patient had intolerable adverse events due to imidafenacin treatment.

## Discussion

The present study is the first to show that imidafenacin may improve the urodynamic parameters and alleviate the urological complications of patients with SCI and neurogenic detrusor overactivity. In this study, we did not find significant improvement in the detrusor pressure, whereas the other parameters were significantly improved. As 23 of 34 patients showed a decreased detrusor pressure (data not shown) and the mean data decreased (Figure 4), it is possible that the small number of patients in this study caused this unexpected result. No patient experienced adverse effects.

A decreased bladder capacity and increased intravesical pressure are the main causes of urological complications in patients with SCI, which lead to poor outcomes such as worse survival due to renal dysfunction and a low quality of life.^1, 2^ Currently, CIC is recommended as the gold standard for SCI bladder management.^1, 4^ However, CIC often becomes difficult to perform in patients with sub-acute SCI and bladder dysfunction, severe paralysis, and/or cognitive problems.^1, 2^ Although these patients are often managed by indwelling catheterization, it should be minimized due to the risk of urinary tract infection and other complications such as uninhibited bladder contraction, a low bladder capacity and low bladder compliance.^2^ Indeed, bladder management with an indwelling catheter worsens bladder compliance over time.^11^

Previous studies have reported positive effects of antimuscarinic agents for neurogenic detrusor overactivity. Evidence has accumulated on the effects of oxybutynin,^20, 21^ trospium chloride,^22^ tolterodine^23^ and propiverine^21^ on decreasing intravesical pressure and improving bladder compliance, although some patients had to discontinue treatment due to typical anti cholinergic adverse events, that is, dry mouth, blurred vision and constipation. In 1976, Thompson *et al.*^20^ reported the effects of oxybutynin in adult paraplegic or quadriplegic patients with neurogenic bladder. In this double-blind, placebo-controlled study, the mean vesical volume was increased, and the intravesical pressure was decreased by oxybutynin.^20^ They concluded that oxybutynin had direct antispasmodic effects on smooth muscles of the bladder.^20^ Stohrer *et al.*^21^ compared propiverine and oxybutynin in patients with neurogenic detrusor overactivity. In their study, the maximum cystometric capacity increased, maximum detrusor pressure during the filling phase decreased, and detrusor compliance improved by both propiverine and oxybutynin.^21^ Despite their positive effects on detrusor overactivity, 63.0% of patients taking propiverine and 77.8% of those taking oxybutynin presented with adverse events.^21^

Imidafenacin and solifenacin are new antimuscarinic agents that have a high affinity for muscarinic receptor subtype M3, which is distributed mainly in the bladder.^16, 24^ Krebs *et al.*^25^ studied the effects of solifenacin in patients with SCI. They reported improvements in the bladder capacity, maximum detrusor pressure and detrusor compliance. In their study, 12% of patients experienced adverse events such as dry mouth and fatigue, and 6% of them discontinued solifenacin treatment because of intolerable adverse events.^25^ Yokoyama *et al.*^24^ compared imidafenacin and solifenacin in patients with overactive bladder. There was no significant difference between the effects or incident rates of adverse events among patients who took these two drugs, except that the duration of dry mouth was significantly shorter in the imidafenacin group than in the solifenacin group at 1 month.^24^ Our results are similar to those of previous reports. No patient had to discontinue imidafenacin treatment due to adverse effects. The bladder volume and bladder compliance significantly increased with imidafenacin treatment. As a bladder capacity <200 ml is a contraindication to CIC,^2^ this change due to the medication has a significant effect on bladder management in those who would recover sufficient hand function for CIC. Imidafenacin treatment along with indwelling bladder catheterization can be an appropriate bladder management option in patients with SCI who are contraindicated to CIC.

Conservative treatment for VUR is controversial. In the clinical practice guideline, the initial and/or temporal use of indwelling catheterization is suggested to be effective for VUR.^2^ In contrast, the long-term use of an indwelling catheter for VUR treatment is reportedly ineffective.^26^ As low bladder compliance due to the long-term use of indwelling bladder catheters can cause VUR,^4, 11^ an increasing bladder volume and compliance are likely to be key for treating VUR. Indeed, Yavuzer *et al.* reported a patient whose VUR was treated with an indwelling catheter and oxybutynin.^27^ In the present study, all 3 patients who recovered from VUR had an increased bladder volume and compliance. As spontaneous recovery can occur,^26^ the effectiveness of anticholinergics on VUR should be further examined.

AD is a potentially life-threatening medical condition that can be triggered by any stimuli below the level of injury in patients with spinal cord lesions at or above Th6. Although the International Standards to Document Remaining Autonomic Function after Spinal Cord Injury defined AD as an increase in systolic blood pressure >20 mm Hg from baseline,^28^ an excessive elevation in the systolic blood pressure, for example, >250 mm Hg, is sometimes observed. As cerebral microbleeds are associated with hypertension,^29^ preventing AD is strongly encouraged during the management of neurogenic bladder in patients with SCI.

Relationships between AD and the urodynamic parameters are inconsistent. In the present study, we did not find any correlation between the incidence of AD and the urodynamic parameters. Giannantoni *et al.*^30^ also reported no significant correlation between AD and the bladder volume, bladder pressure and bladder compliance. In contrast, Huang *et al.*^31^ reported that increases in BP were more significant in patients with severely impaired bladder compliance, i.e., <10 ml cm^−1^ H_2_O, than in those without severely impaired bladder compliance. In the current study, AD was improved in some patients under imidafenacin treatment. It should be noted, however, that symptoms and/or increased blood pressure remained in most patients. Giannantoni *et al.*^30^ found no relationship between AD and the use of antimuscarinics, unless the drugs induced detrusor areflexia. In rats with SCI, increased excitability of bladder afferent pathways was involved in detrusor overactivity, which further results in AD.^32^ Both desensitization of these pathways with chemicals^32^ and the inhibition of bladder contraction with onabotulinum toxin A can reduce AD.^33^ It is possible that imidafenacin alone is insufficient to produce detrusor areflexia and/or suppress bladder afferent pathways.

The limitations of this study include its retrospective design, lack of a control, small number of patients and short follow-up period. The long-term use of indwelling bladder catheters causes the bladder volume to decrease.^11^ Thus, improvement in the bladder capacity found in this study could be caused by imidafenacin treatment and other parameters. However, we still cannot rule out the possibility of spontaneous recovery because of the lack of a control. Indeed, no significant change was observed in bladder compliance with bladder management methods after 1 year from onset.^11^ This could imply that spontaneous recovery would occur within 1 year after the injury. Further study focused on chronic patients is necessary. As we did not evaluate the outcome in terms of the bladder management method after imidafenacin treatment, it remains uncertain whether this treatment can affect the quality of life of patients with SCI. Long-term outcomes, including changes in the urodynamic parameters, bladder complications and bladder management method, should be assessed in the future. It should also be noted that improvement in the urodynamic parameters in the present study was not as much as previously reported; the maximum cystometric capacity increased by about 110 ml with propiverine, 130 ml with oxybutynin^21^ and 75 ml with imidafenacin. It is unknown whether this small change is clinically relevant. Further investigation using a randomized controlled study design is needed to compare imidafenacin with other antimuscarinic agents.

## Data archiving

There were no data to deposit.

## Figures and Tables

**Table 1:** Demographic characteristics

Patient characterics	*n*=34
Age	60±15^a^
*Gender*	
Male	29
Female	5

*Spinal level of injury*	
Cervical	26
Thoracic	8
Lumbosacral	0

Asia impairment scale	
A	2
B	6
C	16
D	10
E	0
Duration of SCI (days)	102±45^a^

*Etiology of SCI*	
Motor vehicle accident	3
Sports	1
Fall	20
Others^b^	10

Abbreviation: SCI, spinal cord injury.

a Mean±s.d.

b Spinal cord infection, spinal caries and post-surgical complication.
